# Deep Electrical Resistivity Tomography for a 3D picture of the most active sector of Campi Flegrei caldera

**DOI:** 10.1038/s41598-019-51568-0

**Published:** 2019-10-22

**Authors:** A. Troiano, R. Isaia, M. G. Di Giuseppe, F. D. A. Tramparulo, S. Vitale

**Affiliations:** 1 Istituto Nazionale di Geofisica e Vulcanologia, sezione di Napoli Osservatorio Vesuviano, Via Diocleziano 328, 80124 Napoli, Italy; 2 Dipartimento di Scienze della Terra, dell’Ambiente e delle Risorse (DiSTAR), Università di Napoli Federico II, Via Nuova Cupa Cintia, 21, 80126 Napoli, Italy

**Keywords:** Volcanology, Geophysics

## Abstract

The central sector of the Campi Flegrei volcano, including the Solfatara maar and Pisciarelli fumarole field, is currently the most active area of the caldera as regards seismicity and gaseous emissions and it plays a significant role in the ongoing unrest. However, a general volcano-tectonic reconstruction of the entire sector is still missing. This work aims to depict, for the first time, the architecture of the area through the application of deep Electrical Resistivity Tomography. We reconstructed a three-dimensional resistivity model for the entire sector. Results provide useful elements to understand the present state of the system and the possible evolution of the volcanic activity and shed solid bases for any attempt to develop physical-mathematical models investigating the ongoing phenomena.

## Introduction

The present research shows the results of an original application of the deep electrical resistivity tomography (ERT) aimed to investigate the central sector of the Campi Flegrei (CF) caldera (Italy).

**Figure 1:**
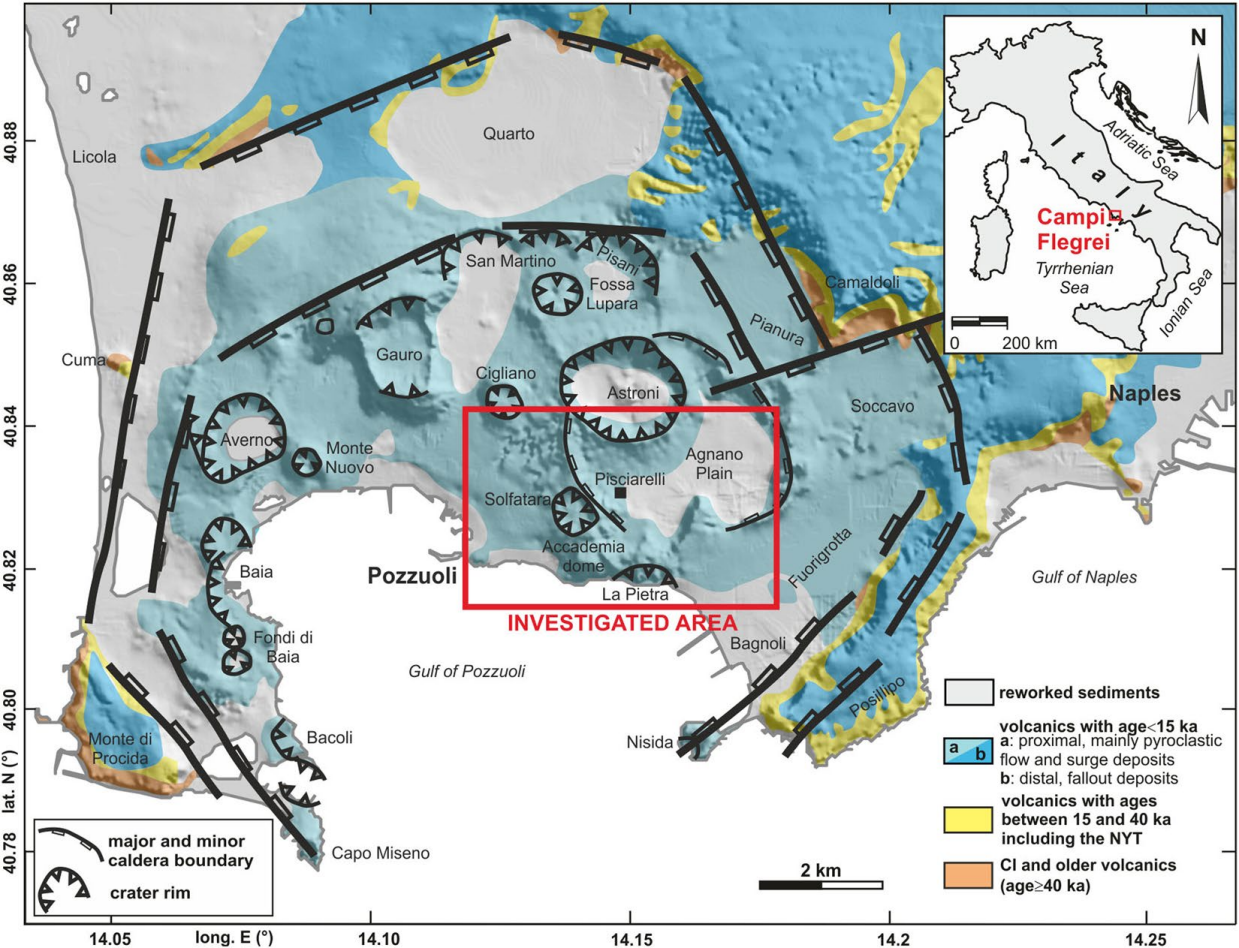
Geological map of the CF area. The red box frames the area resolved by the ERT survey.

The CF caldera (Fig. 1) is one of the most dangerous volcanoes in Europe^1,2^. The most active zone, as for seismicity and gas emission, is currently located in the central sector of the caldera including the volcanic structure of Solfatara, Pisciarelli and Agnano Plain^3–5^.

Intense and recurrent volcanism, culminated in two main caldera forming events, namely the eruption of the Campanian Ignimbrite (39.8 ka)^6^ and that of the Neapolitan Yellow Tuff (15 ka)^7^, marks the CF eruptive history. After this last event, at least 70 mostly explosive eruptions followed, separated by periods of quiescence^8^. The eruptions were mainly of small-medium magnitude, while the few larger have generated minor caldera collapses, as in the case of Agnano-Monte Spina (4.5 ka), which generated the Agnano Plain^1,9,10^. The last eruption occurred at 1538 CE, which led to the formation of Monte Nuovo. According to historical chronicles, different unrest phenomena, such as ground deformation (bradyseism), earthquakes and diffuses degassing^11,12^ preceded this eruption. Phenomena of this kind occurred in the CF before the intense period of volcanism^13^ and even in much more recent times. Since the 1950 CE, three main episodes of ground deformation occurred, the last at 1982–85 CE, with a total uplift of about 3 meters^14,15^. A long period of subsidence up to the 2003 CE followed the last unrest episode. Since 2005 CE, the volcano entered a new phase of unrest, characterized by a strong endogenous degassing, with the extension of the area of carbon dioxide emissions and a slower ground rise with respect to the previous three main ground uplifts^15^. In this context, the most active areas appear to be those of Solfatara maar and the adjacent Pisciarelli fumarole field (Fig. 1).

The eruptive history of the CF is associated with a high level of risk, due to the proximity of the densely inhabited cities of Pozzuoli and Naples. All this made the caldera one of the most studied structures of recent volcanology, subject of a large number of scientific publications as the results of the great effort made by the scientific community in order to achieve a good degree of understanding of the ongoing phenomena. Unfortunately, the current state of knowledge is not complete. The articulated eruptive history originated different vents in the CF, dating back to different epochs and very different stages of evolution^16^. The interaction of these structures with the complex superficial hydrothermal system has concurred to create a scenario in which it is not clear what the driving factor of the current dynamics may be. Presently, several alternative models of CF volcanism coexist in the Literature^17–21^, which in general consider the intrusion of magma in depth as the cause of recent activities, in particular with reference to the crisis of 1982–85 CE. Discordant visions exist on the phenomena in progress to date, on the involvement of a further magmatic contribution from the deep and, in conclusion, on the hazard levels involved. These ambiguities arise from the absence of clear criteria for defining the reliability of the different models, which in turn are linked to a significant lack of structural constraints that would be the basis for developing models going beyond mere theoretical speculation. The nature and geometry of the volcano-tectonic structures that compose the caldera have not been ascertained with certainty, nor the presence of deep magmatic reservoirs or more superficial areas partially melted. Several seismic surveys carried out in the last decades allowed a partial definition of the shallower part of the caldera^22–28^, providing an overall structural definition of the caldera setting, which was reinforced by further geophysical observations carried out with alternative methodologies^29,30^. One of the main unclear issues is connected to the extreme heterogeneity of the shallower part of the caldera, which involves the need for high-resolution geophysical surveys in order to distinguish the subsurface structures associated with different volcanic features.

This paper aims to provide a general view of the CF caldera central sector volcano-tectonic architecture using deep ERT geophysical surveys. The ERT has several characteristics which make it highly suitable for this investigated context. The use of an active source (e.g., a controlled electric current injected into the ground through a source dipole) makes the tomograms reliable even in the highly urbanized environment of the CF. Through totally non-invasive measurements (e.g., the mapping of the induced voltage drops at the surface through a series of receiving dipoles) the detail of the electrical resistivity contrasts present in the subsoil can be reconstructed. The highly sensibility of such parameter to the presence of conductive fluids into the rock matrix makes this technique widely employed to explore volcanically active areas, at shallow depths^31–40^.

Deep ERT is a geo-electrical exploration technique, which peculiarity lays into the physical separation between the source and the receivers^41–47^. This makes it possible, at least in principle, to increase the relative distances between dipoles, which is the element controlling the depth of the geo-electrical imaging, and the realization of irregular arrays, more adequate to explore harsh and urbanized areas. Such possibilities well combine with the technical characteristics of the modern geo-electrical instruments, concurring towards a profitable application of the deep ERT imaging in environments such as that of the CF caldera, where the most relevant structures, even on a shallow scale, are buried at depths of several hundred meters and distributed within a densely inhabited large areas.

## Results

**Figure 2:**
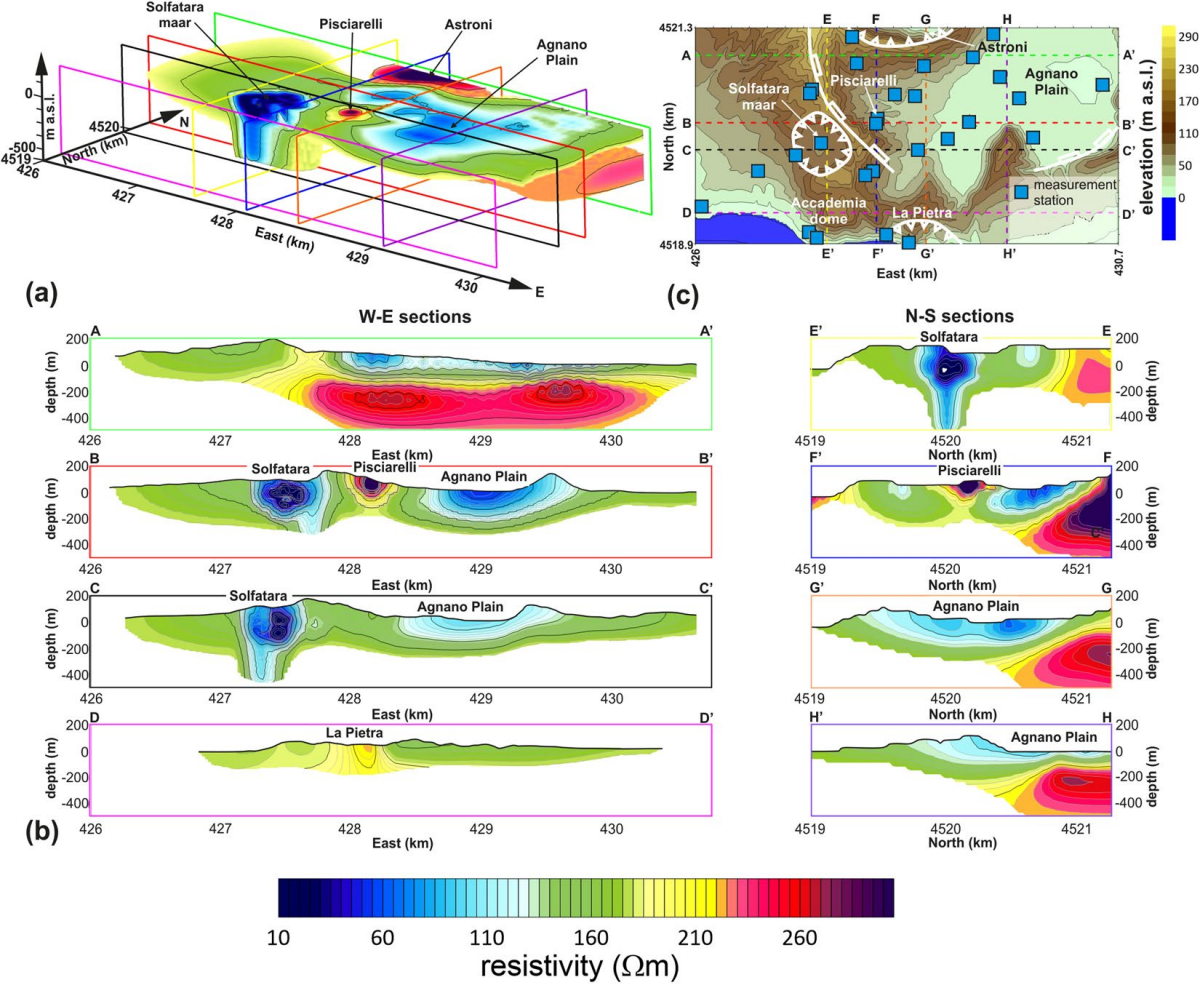
(**a**) Electrical resistivity 3D model obtained through the deep ERT imaging. (**b**) Electrical resistivity cross-sections along selected traces. (**c**) Map of the area resolved by the ERT survey, corresponding to the one framed in the red box of Fig. 1; squares indicate the locations of the sites where the Iris Full Weaver receivers were installed; the colored lines indicate the traces of the selected resistivity cross-sections presented in panel (b); for the geological symbols see Fig. 1.

**Figure 3:**
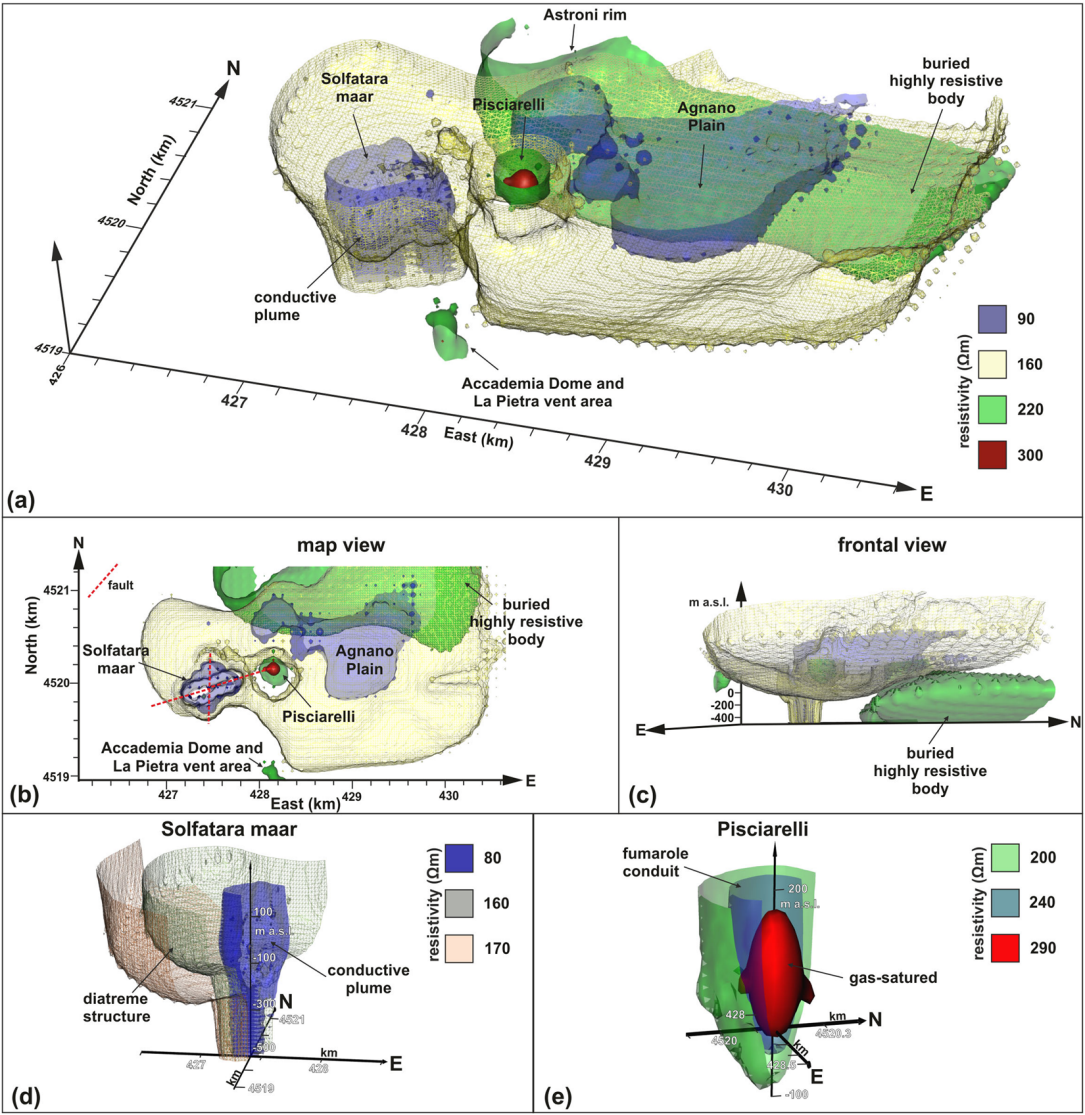
(**a**) Isosurfaces of electrical resistivity extracted from the 3D resistivity model of Fig. 2a; (**b**) map view; (**c**) frontal view; (**d**) detail of the Solfatara structure; (**e**) detail of Pisciarelli structure.

A deep ERT tomography of the central sector of the CF caldera was carried on through the use of several recording stations, acquiring the voltage drops at the end of a series of receiving dipoles. Each station sampled the signal for several days, with a 10 ms sampling rate. At the same time, an almost DC source current was supplied through a power generator and two source electrodes, displaced on the field in a shifted configuration. The adopted source-receivers arrangement was selected considering a preliminary finite element forward modelling. A ERT dataset was extracted from the recorded signals, adopting an original principal component based filtering of the time series and data were inverted through a commercial algorithm, obtaining a three-dimensional model of the subsoil electrical resistivity up to 400 m of depth with respect to the sea level over an area of 2.5 × 1.5 km^2^ of extension. Details about such procedures are presented in the methodological section. The results of the 3D deep ERT imaging are presented in Fig. 2a. Some particularly relevant sections, aligned along with the directions N-S and E-W, are also reported in Fig. 2b (traces are shown in the map of Fig. 2c). An alternative sketch of the 3D model is shown in Fig. 3a–c together with a detail of the large-scale structure of the Solfatara maar (Fig. 3d) and Pisciarelli fumarole field (Fig. 3e), as figured through the deep ERT imaging.

**Figure 4:**
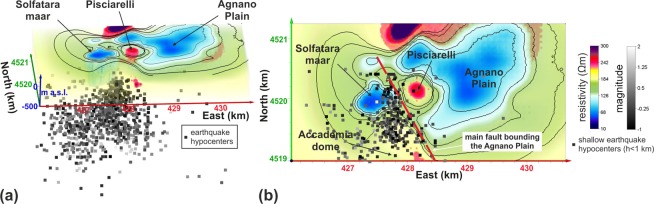
3D-ERT model: (**a**) oblique view showing hypocenters of 2011–2019 CE earthquakes; (**b**) map view showing only shallow hypocenters (h < 1 km).

The electrical tomography allowed us to reconstruct a series of resistivity anomalies by which the main structures buried in the investigated part of the CF caldera can be imaged, and their geometries can be optimally identified. In correspondence of the Solfatara maar, a diatreme structure is quite evidently identified, confirming what previously imaged by geological constraints and shallow electrical tomographies^48^. This structure appears encompassing a conductive plume (Figs 2a,b and 3a,d). In correspondence of the Pisciarelli area, a resistive anomaly of conical shape (Figs 2a,b and 3a,e) is detected, which extends up to about 200 m of depth below sea level. The Agnano Plain corresponds mainly to a conductive anomaly, (Figs 2a,b and 3a,b), which deepens up to about 200 m below sea level. Other significant resistive anomalies correspond to the south-eastern edge of the Astroni crater and the La Pietra vent area (Figs 2a,b and 3a,b). Finally, in order to investigate the relationship between the ground resistivity and the recent seismic activity, we plotted the hypocenters of the earthquakes referred to the 2011–2019 CE interval within the 3D-ERT model (Fig. 4).

## Discussion

The resistivity model presented in Figs 2 and 3 reconstructs with details the main volcanic structures present in the central area of the CF caldera. The model provides, for the first time, an image of this sector in its entirety, reconstructing the reciprocal geometries of the complex volcano-tectonic structures of Solfatara, Pisciarelli, and Agnano Plain. It is worth noting that the more detailed investigations carried out in the past have led to more or less resolved reconstructions of isolated structures. However, a more comprehensive view of all the volcanic features in this sector (or in other parts) of the caldera was previously obtained just in the most extensive seismic or gravimetric models^22,29^. Those latter were characterized by extremely low resolutions, precisely because they were designed for regional scales. The related geophysical surveys require the implementation of complex and very costly field activities, and their re-proposal for scales comparable to the one investigated by the presented electrical tomography is factually not feasible. The scale of the proposed tomographic reconstruction is instead the one that succeeds in providing a clear and well-defined picture of the structures present in the whole caldera sector, as well as evidencing the relationship between those structures and the main superficial fault systems, determining the deeply-rooted formations.

The first electrical anomaly to comment is the one related to the Solfatara volcano. This has already been identified as a maar-diatreme by previous studies, based on surface geological survey and electrical investigations of the volcano crater^31,33,48–50^. The Solfatara picture provided in the model of Fig. 3d represents one of the few examples of three-dimensional reconstruction of the structure of a diatreme presented in the literature. Our results indicate that the Solfatara maar at depth is a fault-controlled structure defined by the straight trends of the resistivity anomaly boundaries. The 3D model viewed in the map (Fig. 3b) clearly shows a complex shape characterized by the intersection of two lineaments NE-SW and N-S directed. The former trend is more developed at depth whereas the N-S trend is well marked in the shallow volume. Indeed, the Solfatara structure is defined by a NE-SW directed conduit, which enlarges upward (200–0 m) with a more equidimensional shape. Structures of such type are typically represented as an inverted cone, or funnel^51^, which can be retrieved in detail from the resistivity model corresponding to the Solfatara area, presented in Fig. 3a,d.

A second very relevant structure identified by the model is the one corresponding to the Pisciarelli area, presently characterized by intense fumarolic emission of CO_2_^52^. In this case, the obtained result has an even more remarkable impact, taking into account the absolute lack of geophysical studies providing reconstructions of this primary interest area. Considering the tomographies presented in Fig. 2a,b, and the sketch presented in Fig. 3e, Pisciarelli sector appears in map view (Figs 2a and 3b) as a circular area (about 250 m of radius) with a relatively high value of resistivity. The correspondence of a high resistivity anomaly extending until at least 200 m from the ground surface (Fig. 3e) attributes a deep origin to the gases permeating the rocks in the area. The gases flow through a conduit likely corresponding to a highly fractured volume resulting by the intersection of major faults such as that bounding the Agnano Plain and the NE-SW trending fault passing for the Solfatara crater^48^. The directions of those faults match the one defining the conduit at base of the Solfatara diatreme.

A remarkable element about the Solfatara-Pisciarelli system is the enlargement of the conductive anomaly across the Solfatara rim, evident in Fig. 3b. Such enlargement likely corresponds to a fluids migration eastwards, that is from Solfatara to Pisciarelli, which was already observed by the comparison between superficial geoelectrical profiles and gravimetric anomaly maps^33^. Numerical investigations devoted to the Solfatara-Pisciarelli system could significantly contribute to confirm this interpretation, gaining help from the geometric definition now obtained.

About the Agnano Plain, it appears as a large conductive area, which is bordered at the north by a resistive mass. The two volumes are separated by a moderately S-dipping discontinuity (Figs 2b and 3b). In the southern sector, different hydrothermal springs are located, and drilling evidence marks the occurrence of highly permeable volcanic and lacustrine deposits, well-consistent with the retrieved higher conductivity value. On the contrary, the northern sector is characterized by a large resistive volume, which was already figured by the inversion of gravimetric data^53^ as an area characterized by a high-density contrast, consistent with the presence of intrusive/lava bodies. Taking into account that (i) results from well log located in the Agnano Plain^54^ indicate how such mass is covered at least by volcanic deposits of 9.5 ka and that (ii) the E-W linear boundary could correspond to a southward moderately-dipping normal fault zone that lowered the southern sector of the Agnano Plain, we suggest that this buried body formed before the Agnano caldera collapse following the 4.5 ka Agnano-Monte Spina Plinian eruption^10^. Furthermore, also considering the resistive area located southeastward the Solfatara volcano, it is possible to note a correspondence with a magmatic mass, again modeled by the inversion of gravimetric data^53^ and exposed along the coast (La Pietra vent area). Finally, a significant correlation exists between the 3D-ERT model and the hypocenters of the 2011–2019 CE earthquakes (Fig. 4). It is worth noting as the shallow hypocenters (with depths lesser than 1 km a.g.l.) are eastward bounded by an NNW-SSE line, likely corresponding to the trace of major fault bounding the Agnano Plain^48^, also well-marked by the resistivity isolines.

### Concluding remarks

The central sector of the CF caldera plays a crucial role in the volcanism of the entire area, especially in the latest unrest phenomena, which have induced to raise to yellow the alert level of the volcano. Deep ERT tomography has proved an appropriate tool for obtaining large-scale images of its main volcano-tectonic features. Actually, beyond the definition of the structural asset, the presented tomography could highlight key-elements about the global CF dynamics, likely driven by the deeper structures present in this sector.

The comprehensive resistivity model of the central caldera was reconstructed with an unprecedented cost/benefit ratio favored by (i) the use of modern instruments; (ii) the application of innovative acquisition criteria and original data processing procedures; (iii) the agility of the field procedures, non-invasive despite the use of an active source; (iv) the electrical resistivity capability to be an unrivalled indicator of the presence of deformation structures that conduit fluids and gases. The resolved detail of the diatreme of the Solfatara, of the degassing area of Pisciarelli, here determined for the first time, of the Agnano Plain and of other significant structures of the sector represent elements of novelty in the framework of the structural knowledge of the caldera, a study area so far somewhat neglected but essential to achieve an adequate basic knowledge of the volcano. All this contributes to giving an insight into the role that modern deep Electrical Resistivity Tomography can play in the context of volcano-tectonic studies, transcending the relevant specificity of the CF.

## Methods

The ERT technique reconstructs the electrical resistivity in the subsoil through the use of an active source^55^. A controlled current is injected into the ground using a couple of electrodes (source dipole), and the induced voltage drop in correspondence with other couples of electrodes (receiving dipoles) is mapped over the Earth surface. The DC source current and the receiving dipoles are displaced on the Earth surface, following the morphology of the environments. Due to the nature of the injected current, inductive and capacitive effects are usually neglected, and the governing equation is:

1 $$\nabla \cdot (-\frac{\nabla v}{\rho })=I\cdot (\delta (\overrightarrow{r}-{\overrightarrow{r}}_{M})-\delta (\overrightarrow{r}-{\overrightarrow{r}}_{N}))$$

where *v* represents the electric potential, ρ is the electrical resistivity of the medium and $I\cdot (\delta (\overrightarrow{r}-{\overrightarrow{r}}_{M})-\delta (\overrightarrow{r}-{\overrightarrow{r}}_{N}))$ are point current sources from two electrodes located at the positions ${\overrightarrow{r}}_{M}$ and ${\overrightarrow{r}}_{N}$^56^.

The measured potential difference is a function of the injected current, dipole geometry and medium response, e.g., its apparent resistivity. Such measurements can be inverted, through a statistical procedure aimed to identify a model of electrical resistivity of the subsoil, which adequately fits the data.

The ultimate commercial, low-cost geo-electrical measuring instruments are capable of acquiring signals sampled at very high frequencies, over a long period at stand-alone receivers. Then, it is encouraged the overtaking of the classic approach to geo-electrical exploration, based on the sequential deployment of the measuring dipoles, which are connected among them through multipolar cables and are displaced on the field in a specific and pre-fixed arrangement. Thanks to the use of physically-separated source and receivers, in fact, surveys composed by a high number of measurements in correspondence of independent receiving dipoles can be nowadays carried out at very moderate cost. Such a possibility well combines with the capability of the modern power generators, which can inject intense currents into the ground. More powerful current sources and the absence of needs of a physical connection between dipoles make it possible, at least in principle, to increase the relative distances between source and receivers, which is the element controlling the depth of the geo-electrical imaging.

### Instruments and field procedures

The deep ERT survey was carried out using Iris Full Weaver^®^ instrumentations, consisting of 12 independent dual-channel voltage receivers, continually recording the signal at 10 ms sample rate. These instruments provided a GPS-synchronized record of the full voltage waveform at the receiving dipoles. The energization current was supplied by an Iris VIP 10000 electrical transmitter, sequentially emitting current pulses of 8 seconds duration. Each pulse was composed of 2 seconds of a continuous current injected with a definite polarity, 2 seconds of stop, 2 seconds of continuous current injected with reverse polarity and 2 seconds of stop. The amplitude of each current step depended on the load, ranging between 5 and 10 A. A further GPS-syncronized Iris full weaver receiver was placed between one of the source electrodes and the VIP transmitter, in order to record the current waveform. To ensure good electrical contact, we used electrodes with a diameter of 20–30 mm and a length of 1 m. Furthermore, in correspondence of each source dipole, three connected electrodes were used to reduce the surface resistance and improve the current injection.

The 12 available Full Weaver recording stations were disposed in the field in continuous acquisition for several days, changing their positions daily (Fig. 1). In correspondence of every measurement site, two orthogonal receiving dipoles of 100 m length were arrayed, aligned in the N-S and W-E directions, respectively. Energizations were carried out in different points every day, displacing a massive injection apparatus composed by the VIP 10000 electric power generator loaded on a Mazda PickUp. A single energization lasted several tens of minutes.

Before of these field activities, the best locations for the receiving and the source dipoles were identified, in order to optimize the survey design to the imaging of the central sector of the CF caldera (Fig. 1). The goodness of every site was evaluated with respect to the data acquisition time. The array geometry was tested realizing forward modeling tests, based on a finite element approach. The Eq. 1 was discretized and solved using the Comsol Multiphysics® commercial software considering specific geometries of the survey, the real morphology of the area and conservative uniform subsoil resistivity models. Essentially, good acquisition geometry of the deep ERT survey has to ensure an optimal source-receivers arrangement. The criteria to satisfy were that (i) sources and receivers have to be sufficiently far each other in order to illuminate structures located at sufficient depths with respect to the ground; (ii) the induced signal, predicted through the modeling, have to be reasonably detectable.

The realized deep ERT array arrangement takes into account source-receiver reciprocal distances varying from 250 m to more than 3.5 km.

### Data analysis and inversion

As previously stated, the depth and resolution of the geo-electrical imaging are controlled by the source-receiver distances and so deeply-located target require a considerable separation between the dipoles. This unavoidably means that induced voltage drops are often extremely low, compared to the instrumental sensitivity and the background noise of both natural and anthropic origin.

The voltage drop recorded during the time in correspondence of an ideal receiver dipole, let us say *v(t)*, can be guessed as the sum of two distinct components. They are the ‘true’ signal *s(t)*, e.g., the part of the voltage induced into the ground by the excitation current, and an independent random component *e(t)*, including all the contribution from any source different from the current supplied for the ERT tomography^57^:

2 $$v(t)=s(t)+e(t)$$

The *e(t)* component can be considered as a noise component affecting the data. The deterministic component *s(t)* presents a cyclic behavior due to the nature of the source. It is characterized by a known frequency and an amplitude θ_0_, which represents the parameter to be estimated.

In the case of the deep ERT survey, a series of records of the voltage drop *v(t)* were realized. A single record consisted in twelve *v(t)* time series, or *tracks*, collected in correspondence of the twelve available Full Weaver during a single injection of the current source, I.

At first, each record was visually explored, windowing each time series in consecutive segments, each one composed of 800 samples and analyzing colored contouring of the recorded voltages as a function of the sample and the window number. An example of such visual analysis is furnished in the Supplementary Materials, with a few comments about such procedure.

The records not showing the required quality, e.g., the ones presenting an evident degradation of the signal-to-noise ratio were subject to an original data filtering procedure. The applied filtering was based on the principal component decomposition^58^, adopted in order to separate *s(t)* from other components, which were less coherent with the deterministic signal. Such an approach emphasizes the multivariate character of the data, e.g., to the fact that all tracks were simultaneously recorded, which is proper of the data acquisition procedure followed. Details about this filtering procedure and examples of the obtained signals are furnished in the Supplementary Materials. In the end, a notable increment of the signal-to-noise ratio was retrieved, also for the cases related to the highest distances between source and receiver. After the application of the principal component filtering, it was possible to obtain adequate signals even in the cases of source-receiver distances of more than 3 km.

Successively, data were inverted through a 3D procedure. The ERTlab3D^®^ commercial software was adopted, based on the inverse algorithm described by^59^. The total dataset was inverted discretizing the subsoil in a tartan mesh composed of 93654 elements and imposing a mixed boundary condition (Dirichlet and Neumann). A starting model characterized by a homogeneous apparent resistivity of 100 Ω*m was considered, and a 5% standard deviation estimate for noise was assumed to invert the data set with a robust inversion. The inversion code finds a regularized solution, looking for the optimal value of the parameter vector **P** and the stabilization parameter α for which minimizing the functional Y(**P**) = χ^2^(**P**) + α**P**^**T**^**RP** results in χ^2^(**P**) =  χ^2^_prior_. The parameters **P** were the natural logarithms of the conductivity of the mesh elements and **R**, the solution roughness, acted as the stabilizing functional. χ^2^_prior_ was equal to the number of data points, and χ^2^ was given by χ^2^ = **(D** − **F(P))**^**T**^**W (D** − **F(P))**, where **D** is the vector of known data values, **F(P)** is the forward solution, and **W** is a data weight matrix. The diagonal elements of **W** were the reciprocals of the data variances, and the off-diagonal elements are zero. This assumed non-correlated data errors. A final rms of about 1.5 were obtained.

Considering the topography in the survey areas and the not regular configuration of the measuring and source sites, complementary reliability checks were carried out, to the aim of evaluating the possible existence of inversion artifacts and assess the resolution of the main electrical anomalies detected by the dERT tomography.

**Figure 5:**
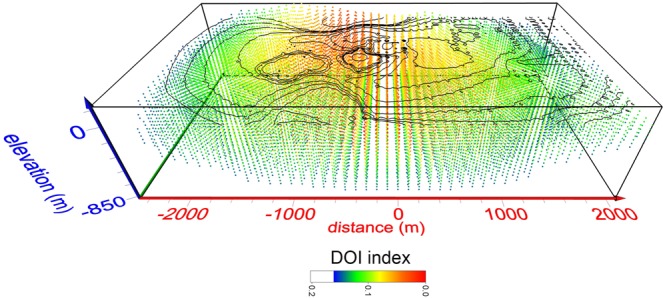
Map of the DOI index values for the dERT model of Fig. 2. Any cells with a DOI index greater than 0.2 were considered less reliable and thus rejected.

First, the depth of investigation (DOI) index was adopted^60–62^. This index requires two further inversions to be carried out, using alternating reference models set to values of one-tenth and ten times the reference model used for the data inversion. The DOI index is then calculated from

$$DOI(x,y,z)=\frac{{m}_{1}(x,y,z)-{m}_{2}(x,y,z)}{{R}_{b}({m}_{01}-{m}_{02})}$$

where m_1_(x, y, z) and m_2_(x, y, z) are the resistivity values in the two final models, and m_01_ and m_02_ are the values of the corresponding reference models. R_b_ are the resistivity values at the base of the two final models, expected to be equal to, or at least very close to, the corresponding value of the reference model. In areas where the final model is well constrained by the data, and the inversion process considered reliable, the DOI index is close to zero. Any cells with a DOI index greater than 0.2^55,61^ were considered less reliable and thus rejected (Fig. 5). The resistivity sections shown in Fig. 2 have been cut out considering such DOI index values.

Secondly, a further reliability check was performed, in order to analyze separately the geometrically smaller significant structures, like those corresponding to the Pisciarelli and La Pietra areas. Such check aims to verify that the collected data were not too sparse to recover these features thoroughly. Their validity was demonstrated by producing totally original tests for each of them, adopting the following procedures.

- The dERT resistivity model (hereafter *R*) was modified in the region corresponding to the checked feature, assigning to such volume uniform resistivity value of 150 Ωm.
- Forward modeling was performed, in order to evaluate the synthetic data that would be generated by such modified resistivity structure (hereafter *R*_*mod*_), conserving the sources and receivers disposition shown in Fig. 2.
- The synthetic data were subsequently inverted, using the same approach adopted for the real data, with the addition of Gaussian noise. An inverted resistivity model was so obtained (hereafter *m*_*mod*_).
- An index is then calculated for each one of the elementary cells composing the inversion mesh grid, from $I(x,y,z)=\frac{{m}_{mod}(x,y,z)-R(x,y,z)}{{R}_{mod}(x,y,z)-R(x,y,z)};$

If the inversion algorithm performs well and the sources-receivers configuration is adequate to correctly recover the feature, the *m*_*mod*_ - *R* discrepancy has to be comparable to the difference between *R*_*mod*_ and *R*. Then, the closer is the I index to the unity, the more the inversion result is valid. A suitable threshold value was assumed, derived from the variance of the added Gaussian noise.

**Figure 6:**
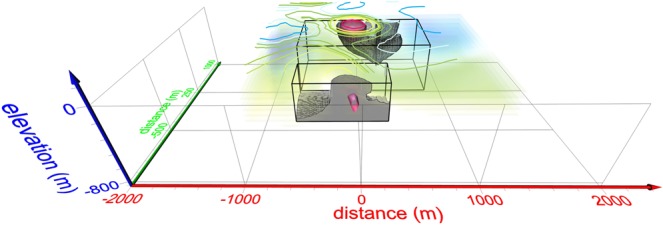
Results of the conformity checks concerning the Pisciarelli and the La Pietra areas, the dERT detected anomalies, which require the higher resolution in order to be correctly imaged. The volumes delimited by the gray surfaces are well constrained by the *I* index.

In Fig. 6 the results of such checks on the Pisciarelli and La Pietra areas are shown.

## Supplementary information

Supplementary Info
