# Publisher Correction: Deep Electrical Resistivity Tomography for a 3D picture of the most active sector of Campi Flegrei caldera

**DOI:** 10.1038/s41598-019-55791-7

**Published:** 2019-12-27

**Authors:** A. Troiano, R. Isaia, M. G. Di Giuseppe, F. D. A. Tramparulo, S. Vitale

**Affiliations:** 1 Istituto Nazionale di Geofisica e Vulcanologia, sezione di Napoli Osservatorio Vesuviano, Via Diocleziano 328, 80124 Napoli, Italy; 2 Dipartimento di Scienze della Terra, dell’Ambiente e delle Risorse (DiSTAR), Università di Napoli Federico II, Via Nuova Cupa Cintia, 21, 80126 Napoli, Italy

Correction to: *Scientific Reports* 10.1038/s41598-019-51568-0, published online 22 October 2019

The original PDF version of this Article contained a missing colour scale in Figure 2. This has now been corrected in the PDF; the HTML version of the paper was correct from the time of publication.

