# Supplementary material for: Deep Electrical Resistivity Tomography for a 3D picture of the most active sector of Campi Flegrei caldera
Source: Sci Rep. 2019 Oct 22;9:15124. doi: 10.1038/s41598-019-51568-0 (PMC6805934; doi:10.1038/s41598-019-51568-0)
Supplement: Supplementary file 1 — Supplementary Info [file 41598_2019_51568_MOESM1_ESM.docx]

**Deep Electrical Resistivity Tomography for a 3D picture of the most active sector of Campi Flegrei caldera**

A. Troiano^1^, R. Isaia^1^, M. G. Di Giuseppe^1^, F. D. A. Tramparulo^1^ and S. Vitale^1-2^

*1) Istituto Nazionale di Geofisica e Vulcanologia, sezione di Napoli Osservatorio Vesuviano, Via Diocleziano 328, 80124 Napoli (Italy)*

*2) Dipartimento di Scienze della Terra, dell’Ambiente e delle Risorse (DiSTAR), Università di Napoli Federico II, Via Nuova Cupa Cintia, 21, 80126, Napoli (Italy)*

Corresponding author: Antonio Troiano ([antonio.troiano@ingv.it](mailto:antonio.troiano@ingv.it))

*Visual analysis of the geo-electrical tracks.*

In order to visually represent the collected data, each track was windowed in consecutive segments, each composed of 800 samples. The contouring of the recorded voltages as a function of the sample and the window number is analyzed. An example of such plot is displayed in Fig. SM1a. The colored strips represents two of the contemporary recorded tracks. Observing the strips, it is possible to evaluate the signal deterioration. For tracks A of Fig. SM1a, corresponding to a low source-receiver distance (a few hundred of metres), the relative strip is composed of a regular sequence of bands, representing the alternation of the two current peaks which compose each individual pulse, separated by the two stop intervals. This means that, for such cases, the voltage drop induced by the current source dominates over the other contributions existing in the signal. When this in not the case, as example when the source-receiver distance increases too much, such a pattern was disturbed and the strips lost their symmetries, as consequence of the component of interest to us being overwhelmed by the other part of the signal. An example of this is the track B of Fig. SM1a.


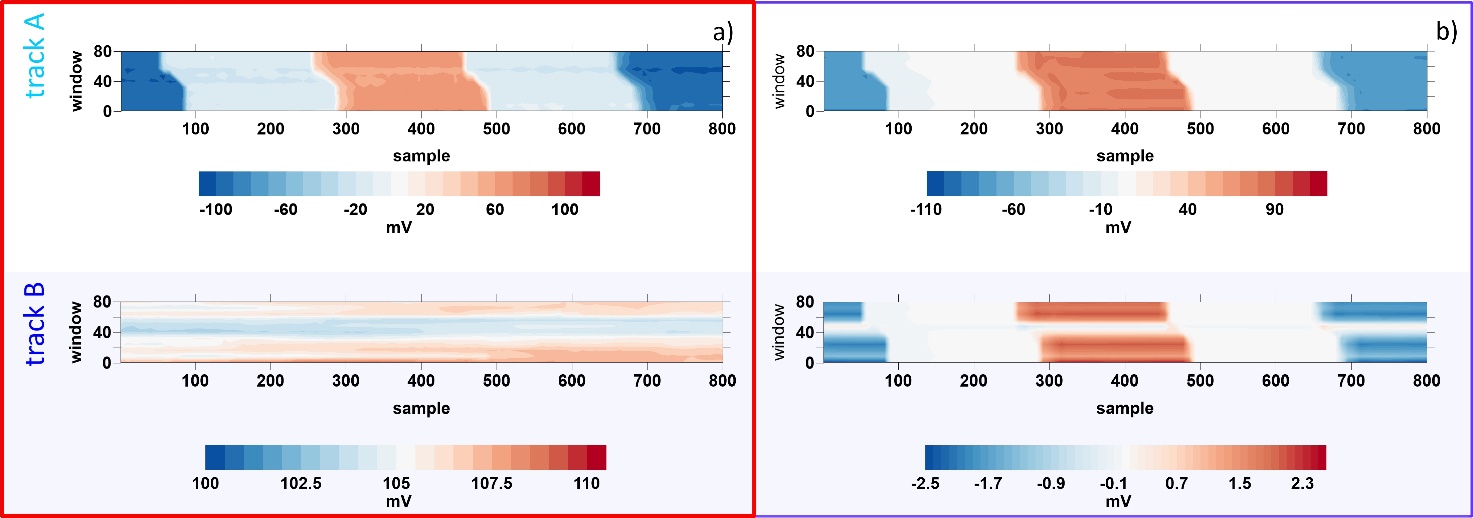


*Fig. SM1. Representation of the recorded (A) and PCA filtered (b) geoelectrical signal. In each colored contour the voltage drops collected at the end of two receiver dipoles is represented, as function of the sample (x-axis) and window number (y-axis).*

*The principal component filtering.*

Details about the principal component statistical technique can be retrieved in Von Storch *et al.* (1999) and references therein. The basis of the principal component analysis relies on the decomposition of an observed dataset X in a simplified structure, which evidenced unknown relationships between the acquired observables. The principal components can be defined as a linear combination of such observables, ordered following a criterion related to the associated amount of information, expressed in terms of variance. It is possible to demonstrate that the principal component is a linear combination of the eigenvectors of the covariance matrix S_X_. Usually, data are often normalized before this decomposition.

The covariance matrix $\boldsymbol{S}_{\boldsymbol{X}}=XX^{T}$ $XX^{T}=WDW^{T}$, can be decomposed as: $\boldsymbol{S}_{\boldsymbol{X}}=WDW^{T}$ $XX^{T}=WDW^{T}$, where W is an orthogonal matrix and D is a diagonal matrix containing the eigenvalues on the main diagonal. However, the well-known SVD decomposition can be introduced, which decompose the X matrix as $X=U\Sigma V^{T}$ $X=U\Sigma V^{T}$, where U and V are unitary matrices and Σ is a diagonal matrix. It follows an alternative form of the covariance matrix S_x_, which can be expressed as $S_{X}=n^{-1}XX^{T}=n^{-1}U\Sigma^{2}U^{T}$ $S_{X}=n^{-1}XX^{T}=n^{-1}U\Sigma^{2}U^{T}$. In other words, the square roots of the Σ matrix coincide with the *D* matrix.

The X matrix can be decomposed through SVD, thus realizing its principal component decomposition and estimating the eigenvectors and eigenvalues of the covariance matrix S_X_. Now it is possible to approximate the X matrix to the one formed by the component related to the contribution of the first two eigenvectors. In this way the principal components can be considered as filter, which has been applied to the recorded data, thus deriving a filtered dataset. The results of such operation are reported in Fig.SM1b, where the filtered tracks are represented, always under colored contour form. The usual estimator applied in the analysis of the geoelectrical signals can be applied on the filtered dataset, retrieving a notable increase in the performances.
